# Cardiac beriberi: morphological findings in two fatal cases

**DOI:** 10.1186/1746-1596-6-8

**Published:** 2011-01-19

**Authors:** Stefania Bello, Margherita Neri, Irene Riezzo, Mohammad Shafie Othman, Emanuela Turillazzi, Vittorio Fineschi

**Affiliations:** 1 Department of Forensic Pathology, University of Foggia, Foggia, Italy

## Abstract

Cardiovascular beriberi is categorized into two main groups, according to its cause: alcoholic and non-alcoholic (dietary). Cardiovascular beriberi can also be divided into a fulminant form (Shoshin beriberi) and a chronic form. Shoshin beriberi is characterized by hypotension, tachycardia, and lactic acidosis and is mainly encountered in non-alcoholic patients in Asian countries, although it has also been seen in alcoholics in Western countries. Due to the complex clinical presentation and to the lack of diagnostic tests, thiamine deficiency is still being missed, especially among non-alcoholics patients. We present two fatal cases of non - alcohol associated cardiac beriberi. An acute myocardial infarction was observed in one case; extensive colliquative myocytolisis (grade 2) was described in the second case respectively. Morphologically, myocardial necrosis and colliquative myocytolysis are the histologic hallmarks of this acute, rare clinical entity. An increase in apoptotic myocytes was demonstrated probably sustaining the cardiogenic shock.

## Background

Thiamine deficiency (beriberi) has two major manifestations: dry beriberi (peripheral neuropathy) and wet beriberi (cardiovascular disease), which include Wernicke-Korsakoff syndrome and lactic acidosis [1]. Deficiency of this vitamin may be nutritional or secondary to alcohol intoxication. Thiamine (vitamin B1) is a cofactor of key metabolic enzymes and thiamine deficiency (TD) may cause alterations in heart metabolism. However, very little is known about the effects of TD on the myocardium. Thiamine is considered a clinically important factor in heart function, and its deficiency has been reported to cause heart failure [2-4]. Due to the complex clinical presentation and to the lack of diagnostic tests, thiamine deficiency is still being missed, especially among non-alcoholics patients [5].

We present two fatal cases of non - alcohol associated cardiac beriberi. Clinical and cardiac morphologic findings are described and discussed.

## Case presentation

### Case 1

#### Clinical findings

A 47 year old - man was admitted to hospital since he had been complaining about cefalea, dizziness, diplopia and oscillopsia for two days. He had a history of gastric resection with a Roux-en-Y reconstruction due to peptic ulcer ten months before. He had no history of alcohol or illicit drug use. Clinical examination revealed normal temperature, blood pressure, heart rate and respiration. He was well orientated; pupils were equal, round and reactive to light, with marked nystagmus on lateral gaze. He exhibited partial bilateral sixth nerve palsy. He moves all four extremities but he had difficulty to stand and ambulate. No other focal neurological deficit was present. Laboratory studies revealed normal serum sodium, serum chloridre, serum bicarbonate, and serum creatinine. The liver function tests showed normal enzymes and serum bilirubin levels with normal albumin. Electrocardiogram and chest radiograph were normal. Magnetic resonance imaging (MRI) and computerized tomography (CT) examinations of the brain were not diagnostic. The spinal fluid cultures were reported as negative. In the suspect of TD, serum thiamine level measurement was programmed but the vitamin was not administered. On the fourteenth day of hospital stay, the patient suddenly experienced chest discomfort, diffuse ST-segment depression in the 12-lead electrocardiogram (ECG) with ST-segment elevation in aVR and rapidly evolving congestive heart failure leading to cardiogenic shock with serious hypotension (60/40 mmHg), hypothermia, intense perspiration, and coma. His arterial blood showed a significant lactic acidosis and he was immediately given bicarbonate (Table 1). The patient developed hemodynamic instability that was refractory to vasoactive drugs followed by unrecoverable cardiac asystole. The day after, the laboratory completed the dosing of the thiamine that resulted equal to 66 nmol/L (n.v. 66 - 200 nmol/L).

**Table 1:** Case 1: the ongoing arterial blood gas results are shown

14^th ^day progressive arterial blood gas results	pH	pCO_2 _mmHg	Lactate mmol/L	HCO_3 _mmol	SBE mmol/L
**16.36 p.m.**	7.032	30	18	7.8	-20.8

**17.35 p.m.**	7.182	46.2	19	16.8	-10.2

**18.02 p.m.**	7.24	57.2	21	21.8	-3.1

**18.12 p.m.**	7.215	47.5	22	24	-3.8

**18.54 p.m.**	7.107	55.2	27	16.7	-11.3

**19.33 p.m.**	7.180	41.0	23	14.7	-12.1

**20.02 p.m.**	7.196	38.4	23	14.4	-12.2

#### Pathological findings

**Figure 1:**
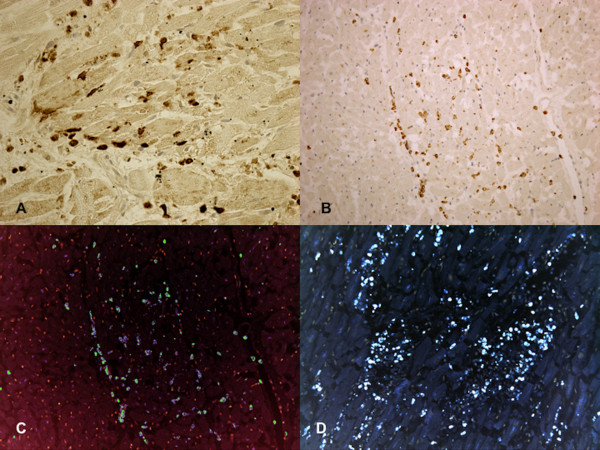
**Myocardial findings in case 1**. **(A) **Infiltration of polymorphonuclear leukocytes (CD15), without fibrin or hemorrhage, into the dead myocardial tissue. **(B) **Immunohistochemical analysis for the phenotypic characterization of the cells revealed a positive reaction to the antibody directed against of polymorphonuclear leukocytes (CD15). **(C) **Confocal laser microscopy: double immunohistochemical reactions for the phenotypic characterization revealed a positive reaction to the antibodies directed against of CD45 (blue reaction) and CD15 (green reaction). **(D) **Myocyte nuclei labelled by TUNEL assay (apoptosis) revealed an intensive, wide, positive reaction (brown nuclei).

At autopsy the body was 175 cm height and 70 kg weight. The heart was normal in size (12×8.5×8 cm) and weight (397 g); the coronary vessels and main branches were normal. Histologically, the heart presented focal myocytes necrosis with massive and diffuse polymorphonuclear leukocytes infiltrate, especially on the anterior left ventricular samples to demonstrate an early myocardial infarction (Figure 1A-D). The heart showed hyper-contraction of the myocell with a breakdown of the whole contractile apparatus with markedly thickened Z-lines and extremely short sarcomeres. This breakdown varied from irregular, pathological and eosinophilic cross-bands consisting of segments of hyper-contracted or coagulated sarcomeres, to a total disruption of myofibrils, the whole cell assuming a granular aspect without visible clear-cut pathological bands. The necrosis was plurifocal, formed by foci ranging from one to thousands of myocardial cells and was found in any cardiac region. Unique bands of 10 to 20 hyper-contracted sarcomeres close to the intercalated disc (paradiscal lesion) were observed too. In addition, the histological sections were analyzed with a confocal microscopy and confirmed the presence of foci of myocardial contraction band necrosis or coagulative myocytolysis visible in all regions of the heart. These pathological changes were formed by paradiscal and pancellular lesions. The latter were formed by segments of hyper-contracted sarcomeres with scalloped sarcolemma. Normal cells around hyper-contracted ones assumed a wavy appearance. No evidence of platelet aggregation or other vessel changes or of interstitial or sarcolemmal alterations existed.

### Case 2

#### Clinical findings

A 43 year - old man, admitted to an hospital due to a severe ulcerative rectocolitis, submitted to total parenteral nutrition (TPN) for 5 days. He underwent abdominal surgery and remained on TPN which was administered without a multivitamin solution. After 3 weeks of receiving TPN he presented hypotension and tachycardia, confusional state and agitation. He complained fever, abdominal pain, nausea, and vomiting; abdominal local tenderness was present. The clinical course quickly worsening; on the fourth week of TNP the man suddenly experienced cardiac arrest and unexplained lactic acidosis: pH7.140; pCO_2 _31.1 mmHg; lactate 19 mmol/L; HCO_3 _11.1 mmol; SBE -18.7 mmol. He was immediately administered vasoactive drugs and bicarbonate. One hour later he died. The dosing of the thiamine resulted equal to 60 nmol/L (n.v. 66 - 200 nmol/L).

#### Pathological findings

**Figure 2:**
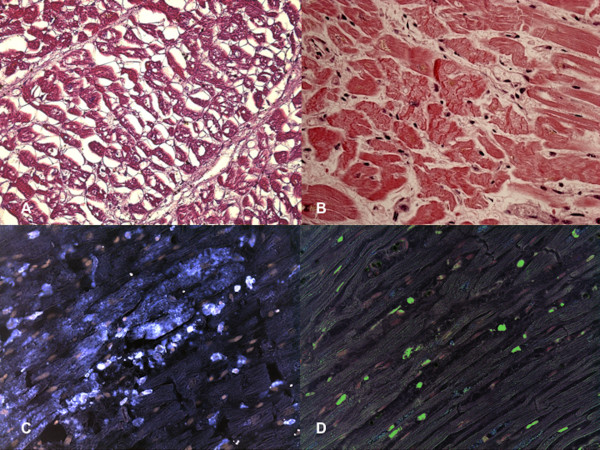
**Myocardial findings in case 2**. **(A) **Colliquative myocytolysis. Perinuclear disappearance of myofibrils with intramyocardial edema resulting in an empty sarcolemmal tube seen in transverse sections. Note the absence of any type of cellular reaction. **(B) **Contraction band necrosis: intense hypereosinophilia of the hypercontracted myocardial cells with rhexis of the myofibrillar apparatus into cross-fiber, anomalous, and irregular or pathological bands. The latter are formed by segments of hypercontracted sarcomeres with scalloped sarcolemma. **(C) **Confocal laser microscopy: CD68 showed a strong reaction in the heart where intense hypereosinophilia of the hypercontracted myocardial cells with rhexis of the myofibrillar apparatus into cross-fiber, anomalous, and irregular or pathological bands is described. **(D) **Confocal laser microscopy: myocyte nuclei labelled by TUNEL assay (apoptosis) revealed an intensive, wide, positive reaction (green nuclei).

At autopsy the body was 168 cm in height and 70 kg in weight. The heart had a normal shape and was normal in size (12×12×6 cm) and weight (410 g). The coronary arteries arose normally; no significant stenosis or thrombotic occlusion of the coronary segments were detected. Plurifocal and interstitial intermyocellular fibrosis due to collagen substitution was evident. Apparently normal nuclei with myofibrillar disappearance producing an increasing vacuolization of myocardial cells until a histologic pattern of empty sarcolemmal tubes without any cellular reaction or signs of healing results. The colliquative myocytolysis was significantly extended, interesting more than 50% of myocells (colliquative myocytolysis grade 3) and was prominent in the subendocardial half of the cardiac wall. Foci of myocardial necrosis characterized by hypercontraction of the myocell with a breakdown of the whole contractile apparatus with markedly thickened Z-lines and extremely short sarcomeres were observed. This breakdown varies from irregular, pathological and eosinophilic cross-bands consisting of segments of hypercontracted or coagulated sarcomeres, to a total disruption of myofibrils, the whole cell assuming a granular aspect without visible clear-cut pathological bands. Myocardium showed focal myocytes necrosis and a typical picture of colliquative myocytolysis with loss of myofibrils paralleled by intramyocellular edema (Figure 2A-D). Myocells with normal nuclei and generalized myofibrillar disappearance producing an increasing vacuolization of myocardial cells without any cellular reaction (grade 2).

#### Immunohistochemical findings

Pathologic features were estimated using histologic sections stained by haematoxylin-eosin (H&E) and thrichrome stain. In addition, immunohistochemical investigation of samples was performed utilizing antibodies anti-CD 45, anti-CD15, anti-CD68 (DAKO, Copenhagen, Denmark) and TUNEL assay (Chemicon, Temecula, CA, USA). We used 4 mm thick paraffin sections mounted on slides covered with 3, amminopropyl-triethoxysilane (Fluka, Buchs, Switzerland). A pre-treatment was necessary to facilitate antigen retrieval and to increase membrane permeability to antibodies. The primary antibody was applied in a 1:600 ratio CD 45, in a 1:50 ratio CD15, in 1:200 ratio for CD68 and incubated for 120 min at 20°C. For TUNEL assay (Apotag Plus Peroxidase In Situ Apoptosis Detection Kit, Chemicon, Tamecula, CA, USA) sections were pre-treated with Proteinase K (Sigma-Aldrich, Buchs, Switzerland) (20 μg/ml) for 15 min, at 20°C; covered and incubated with the TdT enzyme, diluted in a ratio of 30% in reaction buffer for 60 min, at 38°C; put in a coupling jar containing working strength stop/wash buffer, agitated for 15 seconds, and incubated for 10 minutes at 20°C; covered and incubated with anti- digoxigenin conjugate for 30 minutes, at 20°C. The positive reaction was visualized by 3,3- diaminobenzidine peroxidation according to standard methods. The sections were counterstained with Mayer's haematoxylin, dehydrated, cover slipped and observed in a Leica DM4000B optical microscope (Leica, Cambridge, UK).

For semi-quantitative analysis, slides were scored in a blinded manner by two observers (MN, IR). Intensity of immunopositive expression was assessed semiquantitatively in the scale 0-4 as follows: 0 = no immunoreactivity, 1 = mild immunopositivity in scattered cells, 2 = immunopositivity in up to one third of cells, 3 = immunopositivity in up to half cells and 4 = strong immunopositivity in the majority or all cells. In cases of divergent scoring, a third observer (VF) decided the final category. The samples were also examined under a confocal microscope, and a three-dimensional reconstruction was performed (True Confocal Scanner, Leica TCS SPE).

In both cases, myocytes nuclei labelled by TUNEL assay showed an intense, wide, positive reaction. In order to obtain the determination of the fraction of myocytes nuclei labelled by TUNEL, the number of myocytes nuclei per unit area of tissue was determined by counting an average of 10 fields, 1.4 mm2 each, at a magnification of x10 in each area of myocardium sampled. The percentage of apoptotic myocytes nuclei was determined. In case 1, the immunohistochemical study revealed an intensive positive result to TUNEL assay: approximately 38 ± 18% apoptotic cells were observed. CD15 and CD45 positive reactions interesting the anterior left ventricular samples, were observed too.

In case 2, the immunohistochemical study revealed an intensive positive result to TUNEL assay: approximately 54 ± 16% apoptotic cells were observed. CD68 positive reaction was observed around the foci of myocardial necrosis.

In both cases the careful post-mortem examination led to acute cardiac failure due to beriberi as cause of death.

## Conclusions

The presented fatal cases are paradigmatic about cardiac manifestation due to TD. The present report demonstrated the presence of cardiac pathological changes in both cases. Acute myocardial infarction was observed in one case; extensive colliquative myocytolisis (grade 2) was described in the second case respectively. We use the term colliquative myocytolysis to define a progressive loss of myofibrils paralleled by intramyocellular edema [6]. This process starts around apparently normal nuclei with myofibrillar disappearance producing an increasing vacuolization of myocardial cells until a histologic pattern of empty sarcolemmal tubes without any cellular reaction or signs of healing results. This lesion is generally present in the subendocardial half of the cardiac wall. The disappearance of myofibrils especially in the early phases of colliquative myocytolysis is difficult to quantify. Thus, a semi-quantitative evaluation of the extent of this lesion was adopted only when there was total, or almost total, loss of myofibrils. We distinguished the following grades of change: 0-no loss of myofibrils; 1-occasional or small groups of "empty" myocells were seen; 2-less than and 3-more than 50% of myocells in the subendocardial half of the cardiac wall presented this pattern [6].

Cardiovascular beriberi is categorized into two main groups, according to its cause: alcoholic and non-alcoholic (dietary). Cardiovascular beriberi can also be divided into a fulminant form (Shoshin beriberi) and a chronic form. Shoshin beriberi is characterized by hypotension, tachycardia, and lactic acidosis and is mainly encountered in non-alcoholic patients in Asian countries, although it has also been seen in alcoholics in Western countries [5]. Patients may be critically ill with hypotension, hypothermia, and obtundation. Lactic acidosis, oftentimes severe, may occur in thiamine-deficient states. Colliquative myocytolysis is the histological hallmark of congestive heart failure, including acute myocardial infarction in which colliquative myocytolysis expresses a secondary non-ischemic complication involving subendocardial myocardium preserved in infarct necrosis [6]. Few experimental studies showed that thiamine deficiency (during 35 days) decreased the rate of contraction. On the other hand, thiamine deprivation has been reported to cause congestive heart failure [2]. We may only speculate that a decreased contractile compliance by reducing the relaxation phase of the contraction cycle [2] is likely a prime metabolic disorder of the myocardial cell with reduced Ca++ removal from troponine-tropomyosine complex and consequent persistence or prevalence of a contracted state. This is supported by an excess of intramyocellular calcium [7]; strong dependence of systolic and end-diastolic velocity on both number of myocytes and density of myocardial beta- adrenergic receptors [8,9]; increased length with normal length of the sarcomeres of normally sized myocardial cells sampled from failing hearts excised at transplantation and cultured in vitro [10]. In particular, the latter finding suggests an abnormal sarcomerogenesis with a longitudinal apposition of sarcomeres unable to completely relax. An abortive hypertrophy without an increase in myocellular volume thus explain the paradox of an increased heart weight with normal thickness of cardiac walls and myocardial cells in failing hearts [5]. Until it reaches a point of no return, colliquative myocytolysis is likely a reversible lesion, reparable by a re-synthesis of myofibrils [6]. Keep in mind that congestive heart failure in which colliquative myocytolysis predominates, clinical signs (lactic acid formation, low pH, etc.) and symptoms of ischemia are generally present [11,12].

Cardiac beriberi is usually difficult to diagnose because not all cases display the classical signs and there are no specific laboratory tests that can diagnose or rule out the syndrome. Also the measurements of blood thiamine concentration or of the red blood cell transketolase activity miss of specificity and are technically difficult. Thiamine is a cofactor for several essential enzymes in the Krebs cycle and the pentose phosphate pathway, including α-ketoglutarate dehydrogenase, pyruvate dehydrogenase, and transketolase. Thiamine is the precursor for the cofactor of both pyruvate dehydrogenase and α-ketoglutarate dehydrogenase, enzymes that catalyze the oxidative decarboxylation of pyruvate to acetyl-CoA and the oxidative decarboxylation of α-ketoglutarate to succinyl-CoA, respectively. Pyruvate dehydrogenase and α-ketoglutarate dehydrogenase are both key enzymes of the Krebs cycle. A decrease in their activity may lead to increased buildup of toxic intermediates. Lactate accumulation occurs both in the brain and serum because pyruvate cannot enter the Krebs cycle. TD causes an increase in blood and cellular pyruvate concentration and could impair mitochondrial function. Acute heart failure accompanied by ST-segment elevation in patients with a history of alcohol abuse, malabsorption states, malnutrition or eating disorder should prompt the clinician to consider the presumptive diagnosis of cardiac beriberi [11].

Conclusively, there is still an high rate of incorrect ante-mortem diagnosis for wet beriberi, especially in non alcohol - dependent patients. Morphologically, myocardial necrosis and colliquative myocytolysis are the histologic hallmarks of this acute, rare clinical entity. During TD experimentally induced, greater proportion of apoptotic myocytes by TdT-mediated dUTP nick end labeling (TUNEL) and caspase-3 reactivity techniques have been recently described [13]. These results indicate that during TD, reactive oxygen species (ROS) production may be enhanced as a consequence of the installed acidosis [14]. The perturbation in the cardiac myocytes redox balance was responsible for the increase in apoptosis. In our two fatal cases an increase in apoptotic myocytes was demonstrated probably sustaining the cardiogenic shock [15].

## List of abbreviations

CM colliquative myocytolysis; CT computerized tomography; HRP- conjugated antibodies, Horseradish Peroxidase-Conjugated Antibodies; MRI magnetic resonance imaging; TD, thiamine deficiency; TdT enzyme, Terminal Deoxynucleotidyl Transferase Enzyme; TPN total parenteral nutrition; TUNEL, terminal deoxynucleotidyl transferase dUTP nick end labeling.

## Competing interests

The authors declare that they have no competing interests.

## Consent statement

Written informed consent was obtained from the Medical Examiner Department, Court of Justice, for publication of this case report and accompanying images. A copy of the written consent is available for review by the Editor-in-Chief of this journal.

## Authors' contributions

SB and MSO drafted the manuscript. MN carried out the immunohistochemical analysis and performed the microscopic analysis. IR performed the confocal microscopy analysis and performed the microscopic analysis. ET and VF conceived of the study, and participated in its design and coordination and helped to draft the manuscript.

All authors read and approved the final manuscript.

## References

[B1] Astudillo L Degano B Madaule S Sailler L Galinier A Couret B Arlet-Suau E Development of beriberi heart disease 29 years after gastrojejunostomy Am J Med 2003 115 157 158 10.1016/S0002-9343(03)00283-3 12893406

[B2] Oliveira FA Guatimosim S Castro CH Galan DT Lauton-Santos S Ribeiro AM Almeida AP Cruz JS Abolition of reperfusion-induced arrhythmias in hearts from thiamine-deficient rats Am J Physiol Heart Circ Physiol 2007 293 H394 H401 10.1152/ajpheart.00833.2006 17369466

[B3] Roman-Campos D Campos AC Gioda CR Campos PP Medeiros MAA Cruz JS Cardiac structural changes and electrical remodeling in a thiamine-deficiency model in rats Life Sciences 2009 84 817 824 10.1016/j.lfs.2009.03.011 19345230

[B4] Park JH Lee JH Jeong JO Seong IW Choi SW Thiamine deficiency as a rare cause of reversible severe pulmonary hypertension Int J Cardiol 2007 121 e1 e3 10.1016/j.ijcard.2006.08.054 17346820

[B5] Kawano H Hayashi T Koide Y Toda G Yano K Histopathological Changes of Biopsied Myocardium in Shoshin Beriberi Int Heart J 2005 46 751 759 10.1536/ihj.46.751 16157967

[B6] Turillazzi E Baroldi G Silver MD Parolini M Pomara C Fineschi V A systematic study of a myocardial lesion: Colliquative myocytolysis Int J Cardiol 2005 104 152 157 10.1016/j.ijcard.2004.10.051 16168807

[B7] Fineschi V Michalodimitrakis M D'Errico S Neri M Pomara C Riezzo I Turillazzi E Insight into stress-induced cardiomyopathy and sudden cardiac death due to stress. A forensic cardio-pathologist point of view For Sci Int 2010 194 1 8 10.1016/j.forsciint.2009.10.025 19939595

[B8] Riezzo I Di Paolo M Neri M Bello S Cantatore S D'Errico S Dinucci D Parente R Pomara C Rabozzi R Turillazzi E Fineschi V Anabolic steroid - and exercise- induced cardio-depressant cytokines and myocardial β1 receptor expression in CD1 mice Curr Pharm Biotechnol 2011 12 1 10 10.2174/138920111794295792 21050164

[B9] Neri M Bello S Bonsignore A Centini F Fiore C Földes-Papp Z Turillazzi E Fineschi V Myocardial expression of TNF-α, IL-1β, IL-6, IL-8, IL-10 and MCP-1 after a single MDMA dose administered in a rat model Curr. Pharm. Biotechnol 2010 11 413 420 10.2174/138920110791591517 20420568

[B10] Gerdes MA Kellerman SE Moore AJ Muffly KE Clark LC Reaves PY Malec KB McKeown PP Schocken DD Structural remodeling of cardiac myocytes in patients with ischemic cardiomyopathy Circulation 1992 86 426 430 1638711 10.1161/01.cir.86.2.426

[B11] Daly MJ Dixon LJ A case of ST-elevation and nystagmus-when coronary thrombosis is not to blame QJM 2009 102 737 739 10.1093/qjmed/hcp095 19622676

[B12] Kawano H Koide Y Toda G Yano K ST-segment elevation of electrocardiogram in a patient with Shoshin beriberi Intern Med 2005 44 578 585 10.2169/internalmedicine.44.578 16020883

[B13] Gioda CR de Oliveira Barreto T Primola-Gomes TN de Lima DC Campos PP Capettini LDSA Lauton-Santos S Vasconcelos AC Coimbra CC Lemos VS Pesquero JL Cruz JS Cardiac oxidative stress is involved in heart failure induced by thiamine deprivation in rats Am J Physiol Heart Circ Physiol 2010 298 H2039 H2045 10.1152/ajpheart.00820.2009 20304817

[B14] Oliveira MS Floriano EM Mazin SC Martinez EZ Vicente WVA Peresa LC Rossia MA Ramos SG Ischemic myocardial injuries after cardiac malformation repair in infants may be associated with oxidative stress mechanisms Cardiovascular Pathology 2011 20 e43 e52 10.1016/j.carpath.2010.01.012 20185338

[B15] Turillazzi E Riezzo I Neri M Bello S Fineschi V MDMA Toxicity and Pathological Consequences: A Review About Experimental Data and Autopsy Findings Curr Pharm Biotechnol 2010 11 500 509 10.2174/138920110791591481 20420577

